# Genetic Factors of *Campylobacter jejuni* Required for Its Interactions with Free-Living Amoeba

**DOI:** 10.3390/pathogens14060546

**Published:** 2025-05-31

**Authors:** Deepti Pranay Samarth, Asim Z. Abbasi, Young Min Kwon

**Affiliations:** 1 Department of Poultry Science, University of Arkansas System Division of Agriculture, Fayetteville, AR 72701, USA; samarth.deepti@gmail.com; 2 Cell and Molecular Biology Program, University of Arkansas, Fayetteville, AR 72701, USA; asima@uark.edu

**Keywords:** *Campylobacter*, free-living amoeba, environmental survival, host interactions, intracellular survival, genetic factors

## Abstract

*Acanthamoeba*, a free-living amoeba ubiquitous in environmental water, has been considered as the environmental reservoir of certain bacterial pathogens, including *Campylobacter jejuni*, an intracellular human pathogen causing self-limiting gastroenteritis. *Acanthamoeba*-*C. jejuni* interaction mechanisms may help clarify how the otherwise fastidious bacterium *C. jejuni* survives in environmental waters. In this study, we constructed single deletion mutants of *C. jejuni* strain 81–176 for the 10 selected genes (*motAB*, *ciaB*, *kpsE*, *virB11*, *cheY*, *flaAB*, *cstII*, *docB*, *sodB*, and *cadF*) previously shown to be important for the interaction (invasion and intracellular survival) of *C. jejuni* with mammalian hosts. We used a modified gentamicin protection assay to quantify the internalization and intracellular survival of these mutants and the wild type with the two species of *Acanthamoeba* (*A. castellanii* and *A. polyphaga*). Both internalization and intracellular survival were significantly lower for all mutants compared to the wild type with both amoeba strains, except for *ΔcstII* in the internalization assay with *A. castellanii* (*p* < 0.05). The results of this study highlight that the mechanisms used by *C. jejuni* to interact with mammalian hosts are conserved in its interactions with amoeba hosts. This understanding may be useful in developing effective strategies to reduce the transmission of *C. jejuni* to chickens through drinking water.

## 1. Introduction

*Campylobacter jejuni* is a major cause of bacterial gastroenteritis in many countries, including developed countries, responsible for 400 million to 500 million cases of diarrhea each year [1,2,3]. In the United States alone, an estimated 2 million cases of campylobacteriosis occur every year, costing $2.9 billion of economic burden [1,4]. Campylobacteriosis is one of the most reported bacterial illnesses in Europe and affects more than 245,000 individuals yearly [5]. Human campylobacteriosis is mostly a self-limiting acute watery or bloody diarrhea with abdominal cramps and may need hospitalization. Nevertheless, campylobacteriosis can sometimes lead to severe post-infection complications such as Guillain-Barrè syndrome (GBS) and Miller Fisher syndrome (MFS), which could result in some life-threatening consequences. *C. jejuni* infections are thought to induce antiganglioside antibodies in these patients (MFS) by molecular mimicry between *C. jejuni* lipopolysaccharides (LPS) and gangliosides [6]. Even after receiving immunotherapy, approximately 20% of patients with GBS remain severely disabled, with a mortality rate of around 5% [6]. In addition, reactive arthritis and meningitis have also been linked to *C. jejuni* infection [3,7].

*C. jejuni* is a Gram-negative and microaerophilic bacterium from the taxonomical order Epsilonproteobacteria [8]. Except for the cytolethal distending toxin (CDT) and homologs of a type IV secretion system on the pVir plasmid of strain 81–176, *C. jejuni* lacks most of the classical virulence factors present in other gastrointestinal pathogens. Therefore, it has been suggested that the motility and metabolic capabilities of *C. jejuni* could be responsible for its virulence and colonization of the host. However, further research is needed to gain more insights.

Aside from being a commensal resident of the intestinal tract of different warm-blooded animals, various species of *C. jejuni* are also found ubiquitously in the environment [9,10]. *C. jejuni* is often present in chicken caeca in high bacterial loads without causing any obvious clinical symptoms [11]. The thermotolerant species of *Campylobacter* are the most common in human clinical cases, where the majority (over 90%) of these cases are caused by *C. jejuni* [4]. Although *C. jejuni* infections in humans can originate from multiple reservoirs, most cases of campylobacteriosis are often associated with infected poultry carcasses [4,5,12]. Horizontal transmission is the most common way of *C. jejuni* transmission into a chicken flock [9]. Furthermore, during poultry processing, *C. jejuni* originating from intestinal contents can frequently cause cross-contamination of chicken carcasses [13].

Water is considered to be a key contributor to the horizontal transmission of *C. jejuni* in chicken houses [14,15]. Additionally, consumption of contaminated water is linked with many human infections and sporadic outbreaks of campylobacteriosis [14,15]. *C. jejuni* is frequently identified in untreated environmental water [14], which makes recreational water a leading cause of waterborne infections [16,17]. Nevertheless, treated water has also been shown to facilitate the outbreaks of *C. jejuni* infections mainly due to its contamination [18,19,20]. Waterborne *C. jejuni* infections are often underestimated, as such infections are mostly self-limiting and do not require medical care [21,22]. In most of these outbreaks, the numbers of infected patients is smaller in scale as compared to that of foodborne outbreaks, usually less than 5000 cases. Sewage waste, farm animal waste, and wild birds carrying *C. jejuni* commensally in their gut are the primary sources of *C. jejuni* in contaminated water [23]. Contaminated recreational water and seepage of septic sewer into groundwater contaminating drinking water supplies are linked with water-related outbreaks [24] However, it is often challenging to link *C. jejuni* to these outbreaks due to cross-contamination and the inherently sporadic nature of the infections [25,26].

Free-living amoeba (FLA) are gaining recognition as potential environmental reservoirs for many intracellular pathogens, including *C. jejuni* [27,28,29,30]. Microorganisms that resist protozoan grazing are not limited to a particular taxonomic group; instead, FLA hosts a phylogenetically diverse group of microorganisms, including viruses, yeast, and even protists [2,31,32]. The common characteristics among amoeba-resisting microorganisms include their ability to escape the digestion process of amoeba and survive/grow intracellularly [27,33]. Numerous reports support the view that FLA increases the number and virulence of amoeba-resisting bacteria [2,32]. Some bacteria that are well-studied to survive in FLA are *Legionella* spp. [34], *Mycobacterium* spp. [35], *Vibrio cholera* [36], *Coxiella burnetii* [37], *Helicobacter pylori* [26], and *C. jejuni* [38].

FLA are ubiquitously found in the drinking water supply as well as other environmental sources of water and feed on the microorganisms present in the aquatic environment [28,39,40,41,42]). With some exceptions, most members of FLA are non-pathogenic to humans. Therefore, there are fewer efforts to remove them during water treatment. Moreover, FLA remain largely unscathed by water treatment methods [28,43,44].

Many reports substantiate that *C. jejuni* interacts with *Acanthamoeba* spp. in environmental water sources [45,46]. *Acanthamoeba*, commonly present in water and soil, benefits *C. jejuni* by providing it protection from aerobic and physicochemical stress [47,48,49]. However, the molecular mechanisms involved in the interaction between *C. jejuni* and FLA are not well understood. Deciphering such interactions at the molecular level will assist in better understanding *C. jejuni* transmission and will help in developing effective control strategies.

In this study, we have examined the molecular basis for *C. jejuni* interaction with FLA, with a focus on the role of the 10 *C. jejuni* genes with well-known functions for infecting and surviving in mammalian cells. Due to potential variations in different amoeba hosts, two species of *Acanthamoeba* spp., *A. castellanii*, and *A. polyphaga*, were used as model host organisms in the study.

## 2. Materials and Methods

### 2.1. Strains and Culture Conditions

*Campylobacter jejuni* strain 81–176 was used as the wild type and the parental strain to construct 10 deletion mutants of selected *C. jejuni* genes (Table S1). *C. jejuni* strain 81–176 used in the experiments was generously provided by Dr. Michael Slavik, University of Arkansas, Fayetteville, AR, USA. *C. jejuni* strain 81–176 was cultured in Mueller-Hinton (MH) agar or broth at 37 °C under microaerophilic conditions where gas composition is O_2_ (5%), CO_2_ (10%), and N_2_ (remaining balance) and was stored in MH broth containing 15% glycerol at −80 °C. When necessary, antibiotics were added to the media in the following concentrations: trimethoprim (10 μg/mL) and chloramphenicol (6 μg/mL). For all the following experiments, a *C. jejuni* starter culture was prepared by recovering cells from the frozen stock onto MH agar plates with trimethoprim (24 h incubation) and passing heavy inoculum from the agar plates to 5 mL MH broth (16 h incubation). All procedures involving *C. jejuni* strains were conducted according to the protocol approved by the Institutional Biosafety Committee (IBC) at the University of Arkansas.

Two species of *Acanthamoeba* (*A. castellanii* ATCC 50374 and *A. polyphaga* ATCC 30871) were kindly provided by Dr. Kristen E. Gibson (Department of Food Science, University of Arkansas, Fayetteville, AR, USA). The axenic cultivation of the *Acanthamoeba* stains was performed in accordance with the ATCC protocols using peptone-yeast extract-glucose medium (PYG medium ATCC 712; pH 6.5; ATCC, Manassas, VA, USA) with additives (0.4 mM CaCl_2_, 4 mM MgSO_4_·7H_2_O, 2.5 mM Na_2_HPO_4_·7H_2_O, 2.5 mM KH_2_PO_4_, 0.05 mM Fe (NH_4_)_2_(SO_4_)_2_·6H_2_O, 1g/liter sodium citrate·2H_2_O) at room temperature (25 °C) without shaking [50]. When the amoebic trophozoites reached nearly 100% confluency on the bottom surface of the flask, the flask was vigorously agitated by tapping and about 1/10th volume of the suspended amoeba was transferred to a flask with fresh PYG medium for a new culture of *Acanthamoeba*.

*A. castellanii* and *A. polyphaga* were selected based on their broad environmental distribution, distinct physiological traits, and proven effectiveness in *C. jejuni* internalization assays [40,47]. Both species are ubiquitous in natural water sources, where they frequently encounter and interact with a wide range of microorganisms, including *C. jejuni*. Both *A. castellanii* and *A. polyphaga* are well-established in laboratory settings due to their consistent growth under axenic conditions, making them highly suitable for controlled infection assays and mechanistic studies. Additionally, *A. polyphaga* exhibits strong environmental resilience and is often employed to mimic natural host-pathogen interactions under stress-tolerant or variable conditions. Furthermore, both species have demonstrated the capacity to support the intracellular survival and replication of *C. jejuni*.

### 2.2. Construction of Single Deletion Mutants

Ten *C. jejuni* genes, previously confirmed to be important in the interaction of *C. jejuni* with the mammalian host, were selected from previous studies. These genes included *cadF* [51], *cheY* [52], *ciaB* [53], *cstII* [54], *docB* [55], *flaAB* [56], *kpsE* [57], *motAB* [58] *sodB* [59,60], and *virB11* [61].

The complete genome of the *C. jejuni* 81–176 strain (GenBank accession number CP000538.1) was used to design the oligonucleotides listed in Table S2, which were synthesized by Integrated DNA Technologies (IDT, Coralville, IA, USA). The overlapping PCR protocol described by Hansen et al. [62] was used to construct deletion cassettes of the target genes mentioned above. Three DNA fragments (PCR products) were joined by performing overlapping PCR reactions for each gene: approximately 400 bp upstream and downstream regions immediately flanking the target coding sequence (CDS) (amplified from *C. jejuni* strain 81–176 genomic DNA) and the chloramphenicol resistance (Cm^R^) gene amplified from pRY112 [63]. For each target gene, 3 gel-purified DNA fragments were joined together by overlapping extension PCR using Phusion^®^ High-Fidelity DNA Polymerase (New England Biolabs, Ipswich, MA, USA) through overlapping sequences incorporated into upstream and downstream region fragments, creating a deletion cassette. This deletion cassette was used to transform into electrocompetent *C. jejuni* cells by electroporation at 2500 V, and the cells were immediately plated onto an MH agar plate supplemented with chloramphenicol and trimethoprim, followed by incubation for 48 h [64]. Putative deletion mutants were confirmed for the deletion by PCR and Sanger sequencing for all 10 genes. Additionally, natural transformation [64] was performed to transfer the deletion to a fresh background of *C. jejuni* strain 81–176 using genomic DNA of the confirmed deletion mutant. The transformants recovered on MH agar plates supplemented with chloramphenicol were again validated by PCR and Sanger sequencing before making stocks (Figure S1). For *ΔflaAB*, we confirmed a marked decrease in motility as compared to the wild type strain as observed on 0.4% semisolid MH agar plates as described by [65].

### 2.3. Complementation of the Selected Deletion Mutants

Two *C. jejuni* deletion mutants, *ΔcstII* and *ΔcheY*, used in the study were complemented with their respective wild type genes. For *ΔcstII*, the CDS of the *cstII* gene along with its upstream promoter region was amplified from *C. jejuni* 81–176 using Phusion high fidelity DNA polymerase (New England Biolabs), and the primer pair of cstII_comp_F and cstII_comp_R end (Table S2). The primers were designed to include the restriction sites for *SacI* and *KpnI* at the 5’ and 3’ ends, respectively. After purification, the PCR fragment of the *cstII* gene was cloned into the pGEM T Easy^®^ vector (Promega, Madison, WI, USA) using T4 DNA ligase to produce plasmid pGEM-T*cstII*, which was transformed into *E. coli* TOP10 competent cells, and the transformants were screened using the blue-white colony selection method. pGEM-T*cstII* was later used as a source of *cstII* gene insert to construct *pcstII* (pRY108 derivative containing the cstII gene and its promoter). pGEM-T*cstII* was extracted from TOP10 cells with pGEM-T*cstII* using the ZymoPure II Plasmid Midiprep kit and purified before being used in the next step. This step is optional and was performed to streamline the challenges of cloning. Purified pGEM-T*cstII* was digested by restriction enzymes SacI-HF and KpnI-HF. The desired digested fragments were purified after visualizing in agarose gel and gel extraction. pRY108 (shuttle vector, kanamycin resistant) was extracted from *E. coli* DH5 cells (ZymoPURE II Plasmid Midiprep kit; Zymo Research, Tustin, CA, USA). Plasmid pRY108 was also digested by the same set of restriction enzymes, namely SacI-HF and KpnI-HF. Both the *cstII* gene insert obtained from pGEM-T*cstII* and the plasmid pRY108 (digested by restriction enzymes) were ligated using T4 ligase at 16 °C overnight. The ligated pRY108 (p*cstII*) plasmid was transformed into *E. coli* competent cells (Top10) and screened on an LB plate supplemented with kanamycin (50 µg/mL) after 24 h incubation. The insertion of p*cstII* was confirmed by colony PCR, gel analysis following plasmid extraction, and finally by Sanger sequencing.

Using the tri-parental conjugation [66,67] plasmid, p*cstII* was conjugatively transferred into *ΔcstII* with *E. coli* DH5α helping strain containing pRK2013 to create complemented insertional mutants *ΔcstII*/p*cstII*. Tri-parental conjugation was conducted using (1) *E. coli* competent cell Top10 containing p*cstII* (kanamycin resistant), (2) *E. coli* DH5α containing helper plasmid pRK2013 (kanamycin resistant), and (3) *C. jejuni cstII* mutant-*ΔcstII* (chloramphenicol resistant). The potential *ΔcstII*-p*cstII* were screened on MH agar plates containing kanamycin and chloramphenicol. The same process was also used to prepare the complement for *ΔcheY* i.e., *ΔcheY*-p*cstII*. Complementation strains were confirmed by colony PCR and Sanger sequencing using primers that anneal outside of the flanking regions in combination with gene-specific primers. All plasmids used in this study are listed in Table S1.

### 2.4. Internalization Assay

The role of selected genes in the interaction between *C. jejuni* and amoeba was studied using internalization assays. A modification of the gentamicin protection assay described by Dirks and Quinlan [68] was used to perform internalization assays for which centrifuge tubes instead of T-flasks or wells were used at a low centrifugation speed of 600× *g* for 5 min during different washing steps to avoid washing off amoeba cells. Before mixing *C. jejuni* and amoeba cells to create a co-culture for the assay, both amoeba and *C. jejuni* cells were prepared accordingly. The amoeba culture from multiple flasks was pooled and washed three times with Phosphate Buffered Saline (PBS) (Gibco, Waltham, MA, USA) by centrifugation at 600× *g* for 5 min and then resuspended in PBS. Amoeba trophozoites were counted by performing a trypan-blue exclusion assay [69] using a hemocytometer and trypan-blue dye (10×) (Gibco), which allowed us to avoid counting any non-viable amoeba in cell counts. The amoeba cells were visualized for counting by phase contrast microscopy with a 40× magnification in an inverted table-top microscope. Then it was adjusted to a concentration of 1 × 10^7^ amoeba/mL using PBS. *C. jejuni* cells were cultured for 16 h at 37 °C and washed twice before resuspending in PBS. The concentration of *C. jejuni* cells was determined by a spectrophotometer and checked for CFU/mL by plating the serial dilutions on MH agar plates and incubating them for 48 h. Finally, 1 mL of *C. jejuni* (1 × 10^9^ CFU/mL) was mixed with 100 µL *Acanthamoeba* culture (1 × 10^7^ amoeba/mL), resulting in a multiplicity of infection (MOI) of 1000:1 in a 2 mL centrifuge tube. The co-culture was incubated for 3 h at room temperature. A 3 h incubation was chosen based on optimization for maximal *C. jejuni* internalization yield. Longer incubation (4 h) offered no improvement, while shorter duration (2 h) reduced internalization, especially in deletion mutants. The co-culture was then resuspended in 1 mL of gentamicin (200 µg/mL), followed by incubation for 1 h at room temperature. This co-culture was then washed three times to remove any remaining extracellular and attached *C. jejuni*. The wild type *C. jejuni* control was included in each replicate of the internalization assay and plated on an MH agar plate to ensure that gentamicin treatment effectively removed all extracellular *C. jejuni*. For all the wild type *C. jejuni* controls plated after a 1 h gentamicin treatment, we observed zero *C. jejuni* was recovered after 48 h incubation of MH plates. Intracellular bacteria were released by treating this co-culture with 1 mL PYG (ATCC 712) containing 0.3% Triton X-100 (Sigma-Aldrich, St. Louis, MO, USA) for 20 min. After determining the total viable amoeba trophozoite count, the amoeba cell lysate was serially diluted in MH broth, plated onto MH agar plates containing 5 μg/mL trimethoprim, and incubated 48 h at 37 °C under microaerophilic conditions to estimate *C. jejuni* internalization. All the washing steps in this assay and the invasion assay were performed by centrifugation at 600× *g* for 5 min. At all times, an additional control tube containing *C. jejuni*-amoeba co-culture was processed along with the experimental tubes, and the amoeba cell viability and the count were monitored after every step. A *C. jejuni* only control sample was processed alongside to check the efficiency of the gentamicin treatment. We consistently obtained a result of zero CFU from the *C. jejuni*-only control tube after the internalization and invasion assays. Also, a phase contrast microscope (100×) was used to monitor internalization assays. The rate of internalization (ROI), the ratio of internalized *C. jejuni* from recovered amoeba cells, was calculated by first determining the difference between *C. jejuni* CFU from the co-culture (CFU_Co-culture_) and that of the control tube with no amoeba cells (CFU_Control_) and then dividing by the total number of amoeba cells recovered (COUNT_Amoeba_) [68] (1).

ROI = (CFU_Co-culture_ − CFU_Control_)/COUNT_Amoeba_ (1)

### 2.5. 24 h Intracellular Survival Assay

Survival for 24 h inside an amoeba host could provide the required leverage to *C. jejuni* in the harsh environment and support its transmission. The 24 h intracellular survival was examined by a 24 h intracellular survival assay, which was performed in the same way as the internalization assay except for the incubation time of the *C. jejuni*-amoeba co-culture, which was extended to 24 h. After 24 h of additional incubation (intracellular survival period) following a 3 h incubation for internalization, the extracellular *C. jejuni* cells were removed using gentamicin (200 µg/mL for 1 h) followed by Triton X-100 treatment (0.3% in PBS for 20 min). Subsequently, after the Triton-X 100 incubation, all tubes, namely, the control tube (*C. jejuni* without amoeba) and other experimental tubes containing both *C. jejuni* (wild type and mutants) and amoeba, were serially diluted in MH broth, plated onto MH agar plates containing trimethoprim and chloramphenicol (for experimental tubes containing *C. jejuni* deletion mutants), and incubated for 48 h at 37 °C under microaerophilic conditions to evaluate *C. jejuni* 24 h intracellular survival. The rate of intracellular survival (ROIS), the ratio of *C. jejuni* from recovered amoeba cells after a 24 h survival period, was calculated in the same way as ROI (2).

ROIS = (CFU_Co-culture_ − CFU_Control_)/COUNT_Amoeba_ (2)

### 2.6. Statistical Analysis

JMP^®^ genomics 9.0 software program (SAS, Cary, NC, USA) was used for statistical analysis. The statistical significance of differences between groups was determined by one-way analysis of variance (ANOVA) of each pair by using Student’s *t*-test, and the difference was considered statistically significant at *p* < 0.05. The results are expressed as the mean ± standard error. All the experiments were performed at least in triplicate to ensure the replicability of results.

## 3. Results

### 3.1. Selection of the Target Genes for the Study

We have selected 10 *C. jejuni* genes that are responsible for the invasion of and intracellular survival in mammalian cells to gain more insights into *C. jejuni* interactions with one of its environmental hosts, *Acanthamoeba*. The selection of these genes is based on the hypothesis that a certain set of molecular mechanisms utilized by *C. jejuni* to interact with the host cells is conserved between amoeba and mammalian cells. Many previous studies focusing on other bacterial pathogens have shown that there are conserved virulence mechanisms against amoeba and human host cells [42,69,70,71,72]. The 10 selected genes were previously shown to be important for *C. jejuni*-mammalian host interaction through their association with motility, chemotaxis, adhesion, invasion, and intracellular survival [73].

### 3.2. Rationale and Optimization for the Modified Gentamicin Protection Assay

We utilized a modified version of the traditional gentamicin protection assay (GPA) developed by Dirks and Quainlan [68] to determine the internalization and invasion of *C. jejuni* into *Acanthamoeba* cells. Dirks and Quainlan’s version of the modified gentamicin protection assay (mGPA) overcomes limitations of the traditional GPA by using centrifuge tubes instead of cell culture plates, enabling precise control over the co-incubation of amoeba and bacteria, and allowing for *C. jejuni*-only controls. Even actively growing *Acanthamoeba* trophozoites are not firmly attached to the culture base. In traditional GPA, the amoebal monolayer may not be completely stable during wash steps, leading to partial loss of samples and thereby altering results. Furthermore, washes during the mGPA were conducted using low-speed centrifugation rather than by removing and reapplying liquid to a monolayer as performed in traditional GPA. Using mGPA, we achieved lower variability within assay replications. Additionally, our 1 h treatment with 200 µg/mL gentamicin ensured the complete elimination of extracellular *C. jejuni* in the assay, in contrast to other studies that utilized mGPA protocol where they used 100 µg/mL [74,75,76].

We performed a preliminary experiment to optimize the time point for internalization using the mGPA protocol: we co-cultured amoeba and *C. jejuni* for 20 min, 1 h, 2 h, and 3 h, 4 h, and 6 h. After performing gentamicin treatment and lysing amoeba using TritonX treatment, we determined the number of *C. jejuni* cells recovered after plating the TritonX lysate of the amoeba cells. The number of *C. jejuni* cells reached its highest level at 3 h, which did not increase further at 4 h and 6 h co-culture. Therefore, we selected 3 h as the optimal time point and used it in all subsequent internalization assays.

### 3.3. Internalization Assay

**Figure 1:**
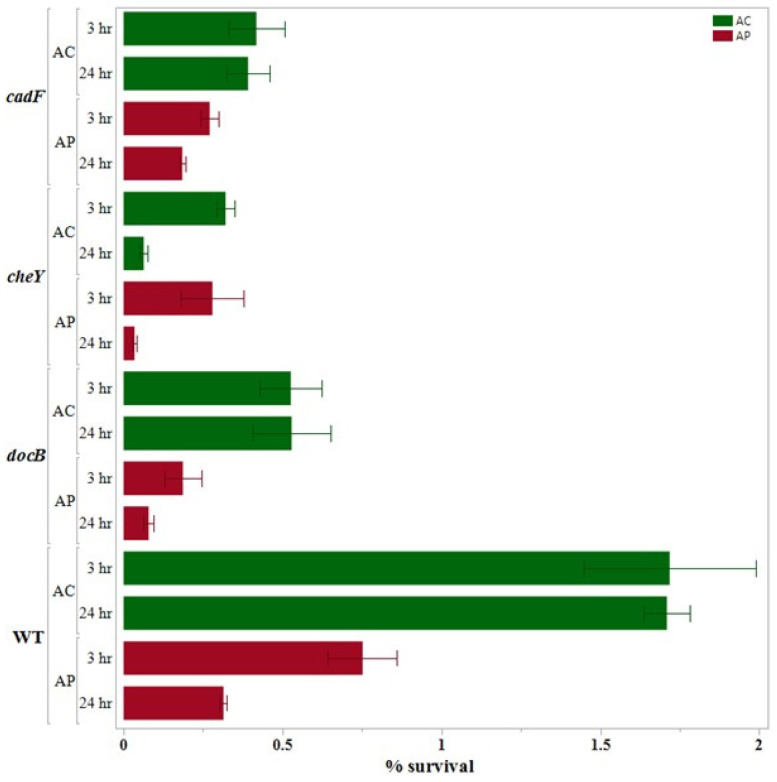
Internalization and intracellular survival (%) of the *Campylobacter jejuni* mutants with a single deletion of the genes responsible for adhesion and chemotaxis as compared to the wild type with *A. castellanii* and *A. polyphaga*. Error bars indicate the standard error of the mean (n = 3).

**Figure 2:**
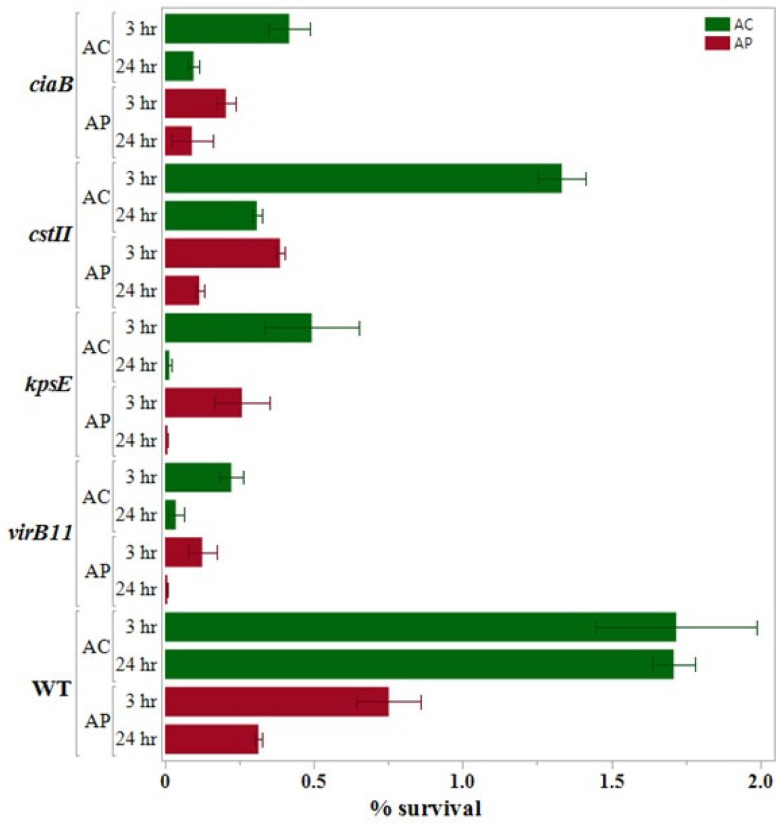
Internalization and intracellular survival (%) of the *Campylobacter jejuni* mutants with the single deletion of the genes responsible for invasion and sialylation of lipopolysaccharide as compared to the wild type with *A. castellanii* and *A. polyphaga*. Error bars represent the standard error of the mean (n = 3).

**Figure 3:**
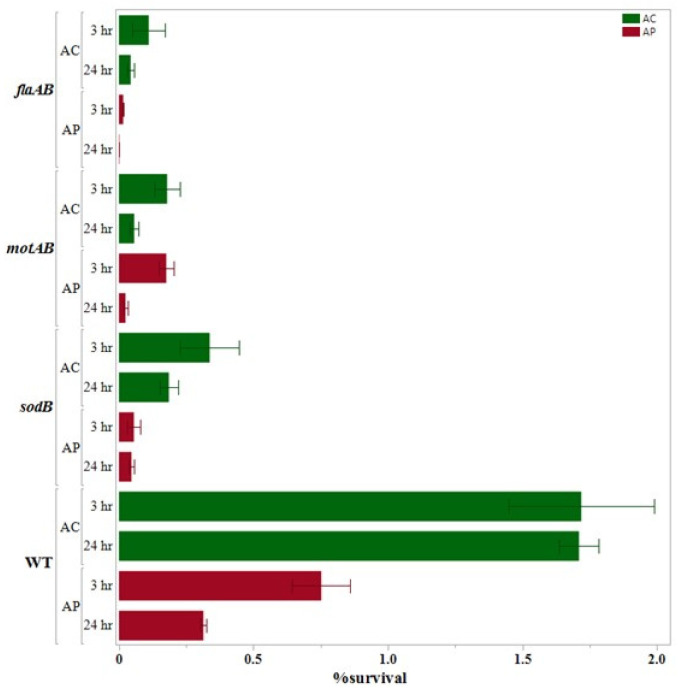
Internalization and intracellular survival (%) of the *Campylobacter jejuni* mutnats with a single deletion of the genes responsible for motility and oxidative stress response as compared to the wild type with *A. castellanii* and *A. polyphaga*. Error bars indicate the standard error of the mean (n = 3).

The results obtained after performing the internalization assays are presented (along with the results of 24 h survival assays) in Figure 1, Figure 2 and Figure 3, which are grouped according to the gene functions: adhesion and chemotaxis (Figure 1), invasion and sialylation of LOSs (lipooligosaccharide; Figure 2), and motility and oxidative stress response (Figure 3).

We noticed that the ROI of the wild type *C. jejuni* was more than double in the *A. castellanii* co-culture (1.72% ± 0.27%) as compared to the *A. polyphaga* co-culture (0.752% ± 0.1085%) at *p* < 0.05. Conversely, in the co-culture with both amoeba hosts, we found that the ROI of the wild type was significantly higher at *p* < 0.05 as compared to all the deletion mutants except *ΔcstII* in *C. jejuni*-*A. castellanii*. In both co-cultures, we found ROI of *ΔcstII* (1.33% ± 0.081 in *C. jejuni*-*A. castellanii* co-culture; 0.3863% ± 0.015% in *C. jejuni*-*A. polyphaga* co-culture) was the highest among the 10 deletion mutants. On the contrary, the ROI of *ΔflaAB* (0.111% ± 0.0605% in *C. jejuni*-*A. castellanii* co-culture; 0.0156% ± 0.0006% in *C. jejuni*-*A. polyphaga* co-culture) was the lowest in both co-culture assays.

### 3.4. 24 h Survival Assay

The successful endobionts that can thrive inside free-living amoeba have evolved to resist protozoan grazing [77]. Few of them use the strategy to avoid degradation by escaping digestion in amoeba cells, often inside non-digestive vacuoles [38,78,79]. The ability to survive for a long time inside amoeba cells might help *C. jejuni* to lie dormant for an extended period and flourish when the time is favorable to *C. jejuni*. We endeavored to examine the long-term survival of *C. jejuni* inside amoeba cells by carrying out a 24 h survival assay performed in a similar way as the internalization assay.

We found significant variation in the results of the 24 h survival assay when the wild type results were compared between the two host species. The percent survival of the wild type after the internalization assay (1.72% ± 0.27%) and after the 24 h survival assay (1.71% ± 0.07%) was almost the same in the *C. jejuni* wild type-*A. castellanii* co-culture. However, in *C. jejuni*-*A. polyphaga* co-culture, the recovery of the wild type *C. jejuni* reduced by nearly half after the 24 h survival assay (0.314% ± 0.108%) as compared to the internalization assay (0.752% ± 0.1085%).

In the 24 h survival assay of both co-cultures (*C. jejuni*-*A. castellanii* and *C. jejuni*-*A. polyphaga*), all mutants showed significantly lower percentages of survival as compared to the wild type at *p* < 0.05. In *C. jejuni*-*A. castellanii* co-culture, among the 10 deletion mutants, we found that ROIS was the highest with *CJΔdocB* (0.529% ± 0.123) and the lowest with CJ*ΔkpsE* (0.0144 ± 0.005). In the *A. polyphaga* co-culture, ROIS was the highest with *CJΔcadF* (0.1844 ± 0.0089), while it was the lowest with *CJΔflaAB* (0.0017 ± 0.00017).

### 3.5. Complementation Assay

In all 10 deletion mutants constructed, the entire coding sequence was precisely replaced with the chloramphenicol resistance gene (with no terminator) in the orientation that would ensure the promoter of the chloramphenicol resistance gene drives the expression of the potential downstream gene(s) in the same operon, thus avoiding any potential polar effect. However, to further validate the role of the genes in internalization and host survival and that the observed phenotype is not due to a polar effect of the deletion, two deletion mutants, *ΔcheY* and *ΔcstII*, were selected and complemented in trans with their respective genes along with their promoters.

**Figure 4:**
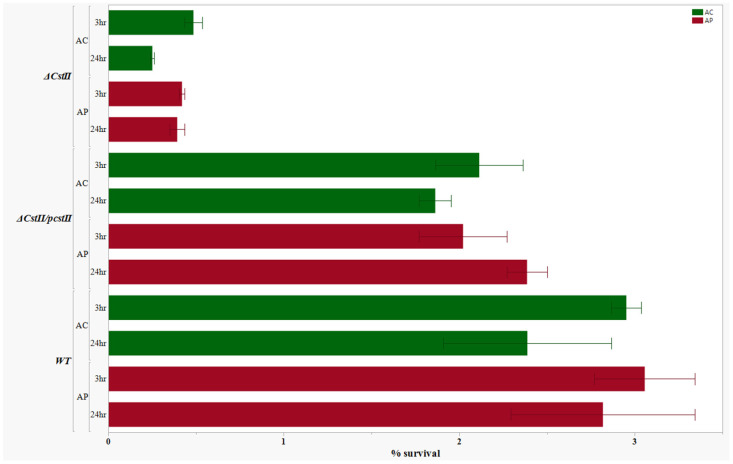
Complementation assay for the *Campylobacter jejuni ΔcstII*. Comparison of internalization and 24 h intracellular survival (%) of the mutant without (*ΔcstII*) and with the complementation (*ΔcstII*/p*cstII*) and the wild type with *A. castellanii* and *A. polyphaga*. Error bars indicate the standard error of the mean (n = 3).

**Figure 5:**
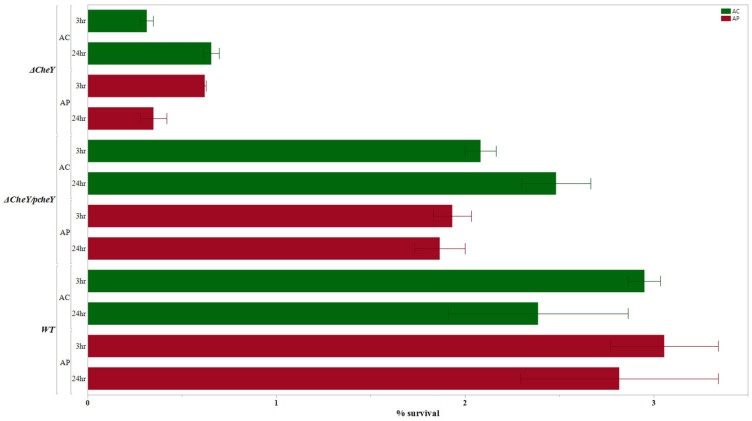
Complementation assay for the *Campylobacter jejuni ΔcheY*. Comparison of internalization and 24 h intracellular survival (%) of the mutant without (*ΔcheY*) and with the complementation (*ΔcheY*/p*cheY*) and the wild type with *A. castellanii* and *A. polyphaga*. Error bars indicate the standard error of the mean (n = 3).

After performing an internalization assay and 24 h survival assay, the results of the assays were compared among the deletion mutant, complemented mutant, and the wild type for both *cheY* and *cstII* genes. We found that the mutants *ΔcheY* and *ΔcstII* had reduced capability to internalize and survive within selected amoeba strains. However, after these mutants were complemented with the *cheY* and *cstII* genes using the pRY108 plasmid, the complemented mutants, *ΔcstII*/p*cstII* and *ΔcheY*/p*cheY*, restored their capacity to internalize and survive to levels closer to those of the wild type *C. jejuni* for both *Acanthamoeba* species (Figure 4 and Figure 5). The results from the complementation assay for these two selected genes strongly suggest that our approach for construction of deletion mutants reliably reveals the phenotypic changes due to the deletion of the target genes of interest.

## 4. Discussion

FLA is known to be an environmental host for many organisms smaller than them in size [2,32]. The amoeba grazes and depends on these organisms for their food [80] (Horn & Wagner, 2004). A number of studies have emphasized the role of unicellular eukaryotes, particularly FLA, on the survival and dissemination of many waterborne bacterial pathogens [77]. FLA grazing is resisted by many bacteria [33,38,81]. Many of them can survive intracellularly, including *C. jejuni* [38], *Mycobacterium avium* [82], *Legionella pneumophila* [83] (Ghosh et al., 2024), *Listeria monocytogenes* [84], *Francisella tularensis* [85], and *Vibrio cholera* [36].

Individual cases and sporadic outbreaks of campylobacteriosis often caused by contaminated water, raise questions on the mechanism by which *C. jejuni* survives in the environment. Despite being fastidious in nature when cultured under laboratory conditions, *C. jejuni* can survive in the environment for long periods [86]. Casalino et al. [87] reported that 43.5% of sampled laying hens housed in various rearing systems—barns, cages, and aviaries—were positive for *C. jejuni*, with 17.5% of the isolates exhibiting multidrug resistance, underscoring the bacterium’s persistence and adaptability in poultry environments. FLA in environmental water could protect it from inhospitable environmental conditions and may support its survival under atmospheric oxygen tension. The association of *C. jejuni* with FLA and algae in environmental water has been described for more than a decade through many studies [45,47,49,88]. *Acanthamoeba* has emerged as a valuable model for studying the interactions between bacteria and eukaryotic cells due to its ability to mimic key aspects of phagocytosis and intracellular survival. Its natural role as a predator of microbes makes it particularly suited for investigating bacterial pathogenicity and host-cell adaptation mechanisms [30,89]. Snelling et al. [49] indicated that FLA, which contains *C. jejuni* intracellularly, can transmit *C. jejuni* to chickens in chicken houses, and this increases the risk of transmission from drinking water. Olofsson et al. [90] have demonstrated that the presence of FLA increases the survival efficiency of *C. jejuni* in milk and orange juice, which also could add to cases of campylobacteriosis. Co-cultures with *Acanthamoeba* are even recognized to enrich the low concentration of *Campylobacter* spp. from environmental samples without the dependency on microaerophilic conditions, which is a basic requirement for culturing *C. jejuni* in the laboratory [91,92]. Moreover, evidence suggests that the presence of *Acanthamoeba* helps *C. jejuni* to increase tolerance towards chemical stress commonly occurring in the environment, including chlorine used to treat drinking water [48,93]. There are scientific reports on the interactions between *C. jejuni* and *Acanthamoeba* with mixed conclusions [46] (Vieira et al., 2015). Most studies support the idea of intracellular survival of *C. jejuni* inside *Acanthamoeba* cells [38,45,49,74,88,94]. Axelsson-Olsson et al. [45] have provided a microscopic image of *A. polyphaga* infected by *C. jejuni* residing inside its vacuole, which was obtained after the co-culture of both organisms. Olofsson et al. [38] showed that *C. jejuni* can actively invade *A. polyphaga*, persist, and replicate in the vacuole. Some reports accept the advantages of the *Acanthamoeba* co-cultures on *C. jejuni* survival [95,96,97]. Xuan Thanh Bui et al. [96] suggested that *Acanthamoeba* cells reduce the oxidative stress of *C. jejuni* when it is in the surrounding of FLA, as it depletes oxygen from the surrounding. In both ways, intracellularly or in *Acanthamoeba’s* proximity, the association between *Acanthamoeba* and *C. jejuni* can significantly affect *C. jejuni* transmission to both humans, both directly and through sources that could eventually increase incidences of *C. jejuni* infections in humans. This creates an urgency to study different aspects of this interaction, especially molecular interactions, as we have little knowledge about it.

Human macrophages are analogous to *Acanthamoeba* cells [98] in their (1) eukaryotic nature, (2) morphology and structural features [41], (3) amoebic invasive properties including phagocytosis [38,78,99], and (4) similarity in mechanisms at the transcriptional, post-transcriptional, and cellular levels [100,101,102,103,104,105]. These similarities are demonstrated in studies focused on *Staphylococcus aureus* [106] and *Legionella pneumophila* [104,107,108]. Many eukaryotic processes are highly conserved through evolution, including phagocytosis, vesicle traffic, and endosomal-lysosomal degradation [109]. *C. jejuni* can infect and persist intracellularly inside vacuoles in both mammalian and *Acanthamoeba* cells. These point towards the possibility that bacteria could carry out their infection in both hosts in a similar fashion [55,101,110,111]. There is considerable research on the genetic basis of interactions between *C. jejuni* and mammalian cells, including human epithelial and macrophage cells [53,55].

In this study, we chose two strains of *Acanthamoeba* that were different in their cell sizes [112], *A. casetallani* (12 to 35 μm trophozoite) and *A. polyphaga* (30 to 60 μm trophozoite), to examine their ability to harbor *C. jejuni*. Choosing two strains of the most commonly isolated FLA from environmental water sources [39,113] would allow a broader perspective on their interactions. *Acanthamoeba* is a suitable choice to study interactions of *C. jejuni* with FLA for a number of reasons: (1) they can be grown axenically (without using bacteria as amoeba feed), (2) they can significantly prolong *C. jejuni* viability in low nutrient conditions [45], and (3) it has been reported to protect some bacteria from halogen disinfectants [49] (Snelling et al., 2008).

Typically, the survival of *C. jejuni* inside a host cell is examined using the GPA [114]. We tried to use this method to study the internalization and intracellular survival potential of the deletion mutants to survive inside amoeba host cells. Unfortunately, we experienced significant variations between different replicates of GPA due to the suspension nature of the *Acanthamoeba* cell culture. As the cells are loosely attached to the base of the culture flask, we lose cells during the wash steps. This caused variation in the count of *Acanthamoeba* cells and eventually led to inconsistency in recovering *C. jejuni* cells [76]. We found that using mGPA developed by Dirks and Quinlan [68], we were able to eliminate the problem of experimental variation. Additionally, by counting *Acanthamoeba* cells after the assay is performed, we know exactly how many *Acanthamoeba* cells are remaining, which helps us to calculate the survival percentage of recovered *C. jejuni*. mGPA was conducted in centrifuge tubes instead of culture flasks, and a low centrifugation speed of 600× *g* (to reduce the cell damage by centrifugation) for 5 min was used for washing to avoid washing off the amoeba cells. Furthermore, at the beginning of the experiment, we counted the number of *Acanthamoeba* and *C. jejuni* cells to be used in the experiment. *Acanthamoeba* cell number and cell viability were monitored at each step, which helped us eliminate the experimental variation.

We carefully chose a 3 h incubation time in the internalization assay after experimental optimization to allow maximum internalization of *C. jejuni*. Allowing a longer incubation period (4 h incubation time) did not help in increasing the number of *C. jejuni* in the internalization assay; whereas, we obtained a lower number of *C. jejuni*, especially when using deletion mutants, when a 2 h incubation time was selected. We also performed mGPA with an extended 24 h period to monitor *C. jejuni* survival in *Acanthamoeba* cells. Our results are in line with those presented by Axelsson-Olsson et al. [45], which also support the survival of *C. jejuni* in *A. polyphaga* for up to 48 h. The survival of *C. jejuni* for 24 h would indicate the ability of *C. jejuni* to persist in amoeba cells for a duration that could be significant enough to affect *C. jejuni* transmission and could play a major role in *C. jejuni* epidemiology. *C. jejuni* might use different sets of mechanisms to survive intracellularly.

We found that there is a variation among two *Acanthamoeba* strains in their ability to internalize *C. jejuni* cells. *A. castellanii* was better at internalizing *C. jejuni* cells as compared to *A. polyphaga*. A similar tendency was also reflected in the 24 h survival assay, where *C. jejuni* was able to survive intracellularly for a longer period in *A. castellanii* as compared to *A. polyphaga*. We know that the two *Acanthamoeba* strains differ in their cell size, and there could be differences in their bacterivorous ability. Currently, the possible reasons for this variation are unknown, and more research is needed to improve the understanding of different factors that could contribute to the bacterivorous ability of *Acanthamoeba* strains. Nasher and Wein [74] have observed a difference in the number of *C. jejuni* cells recovered after *A. polyphaga* and *A. castellanii* internalization assays. However, they found that *A. polyphaga* was slightly better in C. jejuni internalization as compared to *A. castellani*.

We found that all the selected ten *C. jejuni* genes that are important in mammalian cell internalization or intracellular survival are also important in the interaction with *A. castellanii*, except for *ΔcstII*, which had lower ROIs as compared to the wild type but was not significant (*p* > 0.05). The same trend was observed in the interaction with *A. polyphaga*, where all the genes appear to be important for the *C. jejuni*-*A. polyphaga* interaction.

Nasher et al. [18] also observed similarly reduced internalization with flagella gene mutants (*flaA* and *flaB*) in *C. jejuni* strain 11168 in an internalization assay and upregulated *flaB* in a *C. jejuni* RNAseq transcriptome analysis from *C. jejuni*-*Acanthmaoeba* internalization assay, underscoring the importance of *C. jejuni* flagella in *C. jejuni*-*Acanthamoeba* interactions. Nasher and Wein [75] revealed that non-motility-related properties of the flagella are essential for interactions with *Acanthamoeba castellanii* and that O-linked glycosylation of flagellin aids in the recognition, capture, and phagocytosis. Nasher et al. [115] and Nasher and Wein [75] have used a modified GPA version where culture is used, whereas mGPA has employed centrifuge tubes and slight centrifugal force to perform wash steps.

Nasher and Wein [94] emphasized the crucial role of *C. jejuni* invasion antigens (Cia proteins) in host-pathogen interactions, demonstrating that the *ΔciaB* mutant is cleared and digested more rapidly compared to the wild type strain. Vieira et.al. [76] examined a different genetic component, the CmeABC multidrug resistance (MDR) efflux pump, and found that contributes to bacterial survival within amoeba. The role of the CmeABC efflux pump in colonization is also conserved when colonizing *Acanthamoeba* and chicken hosts [116].

The results of this study highlight the conserved mechanisms of *C. jejuni* internalization and intracellular survival between mammalian and amoeba hosts. The involvement of multiple genes in the interaction with amoeba indicates that *C. jejuni* utilizes multiple mechanisms to invade and survive in amoeba. The results of the intracellular assay also indicates the possibility that *C. jejuni* may seek shelter in amoeba to survive in the chicken house water supply. However, due to the complexity of host-pathogen interactions, we encourage expanding the study by selecting additional genetic factors and testing a broader range of *C. jejuni* strains to gain a more comprehensive understanding of the mechanisms involved.

*Acanthamoeba* is not only considered a transient host in the environment where it is also termed a “Trojan horses of microbial world” [70] but also suspected to be a training ground where these endosymbiotic bacteria improve and develop mechanisms for infecting higher animals [98,105,117,118,119]. This theory can be supported by our study, as we found a similarity in the mechanism by which *C. jejuni* infects both amoeba and mammalian cells.

Our study is an effort to shed light on the molecular mechanisms in *C. jejuni-Acanthamoeba* interaction. A better understanding of these processes will help to develop novel strategies and targets for vaccines and antibiotics against *C. jejuni*. Further studies are needed to obtain more detailed insights into the role of free-living amoeba as hosts in *C. jejuni* epidemiology.

## Data Availability

All data from the study are presented in the manuscript. Any additional information from the study will be made available upon request to the corresponding author.

## Supplementary Materials

The following supporting information can be downloaded at: https://www.mdpi.com/article/10.3390/pathogens14060546/s1, Figure S1: PCR confirmation of the deletion mutants; Figure S2: Microscopic image of the actively growing culture of *Acanthamoeba castellani*; Table S1: Bacterial strains and plasmids used in the study; Table S2: Oligonucleotides used in the study.

## Abbreviations

The following abbreviations are used in this manuscript:

GBS	Guillain-Barrè syndrome
MFS	Miller Fisher syndrome
LPS	Lipopolysaccharides
CDT	Cytolethal distending toxin
FLA	Free-living amoeba
GPA	Gentamicin protection assay
mGPA	modified gentamicin protection assay
LOG	Lipooligosaccharide
MH agar	Mueller-Hinton agar
PYG medium	Peptone-yeast extract-glucose medium
MOI	Multiplicity of infection
ROI	Rate of Internalization
ROIS	Rate of Intracellular Survival
MDR	Multidrug resistance
