# Supplementary material for: Genetic Factors of Campylobacter jejuni Required for Its Interactions with Free-Living Amoeba
Source: Pathogens. 2025 May 31;14(6):546. doi: 10.3390/pathogens14060546 (PMC12195619; doi:10.3390/pathogens14060546)
Supplement: Supplementary file 1 [file pathogens-14-00546-s001.zip › pathogens-3616843-supplementary.pdf]

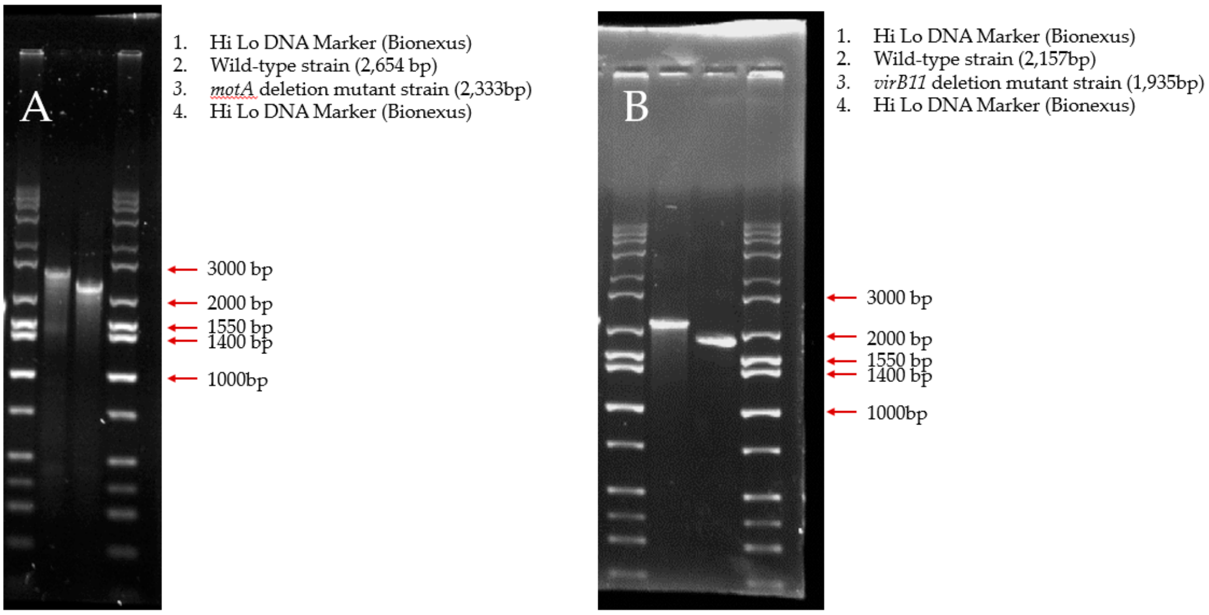

Figure S1. PCR Confirmation of the deletion mutants. (A) *motAB* gene and (B) *virB11* gene. PCR products were amplified from the wild-type strain and respective deletion mutant of *C. jejuni* strain 81-176 using the primers annealing to the regions outside the upstream and downstream homology regions. The PCR products were separated on an 1.2% agarose gel.

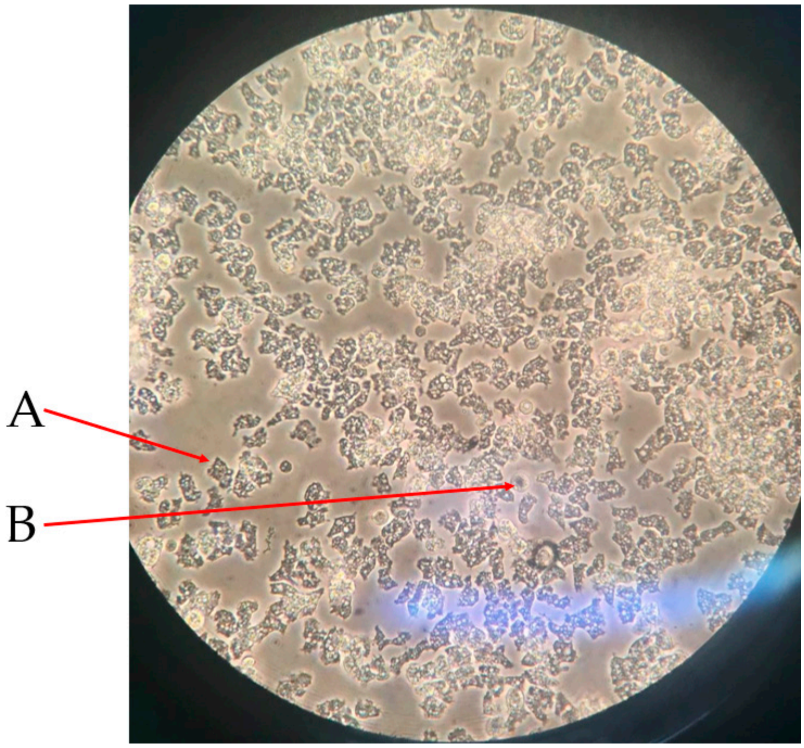

Figure S2. Microscopic image of the actively growing culture of *Acanthamoeba castellani.* The image was viewed in a Phase contrast microscope (Nikon Eclipse 50i clinical microscope) at a 10X magnification. The image shows (A) trophozoites (vegetative amoeba cell) and (B) cyst form of *A. castellanii.* Higher proportion of trophozoite to cyst ratio suggests a healty cell culture. Trophozoites transform into a resistant cyst form before death, while favourable conditions permit vegetative growth as trophozoites.
